# Clinical decision support for gastrointestinal panel testing

**DOI:** 10.1017/ash.2024.15

**Published:** 2024-02-08

**Authors:** Nadia T. Saif, Cara Dooley, Jonathan D. Baghdadi, Daniel J. Morgan, KC Coffey

**Affiliations:** 1 Department of Epidemiology and Public Health, University of Maryland School of Medicine, Baltimore, MD, USA; 2 Department of Medicine, Veteran’s Affairs (VA) Maryland Healthcare System, Baltimore, MD, USA

## Abstract

**Objective:**

This study aimed to assess the impact of clinical decision support (CDS) to improve ordering of multiplex gastrointestinal polymerase chain reaction (PCR) testing panel (“GI panel”).

**Design:**

Single-center, retrospective, before-after study.

**Setting:**

Tertiary care Veteran’s Affairs (VA) Medical Center provides inpatient, outpatient, and residential care.

**Patients:**

All patients tested with a GI panel between June 22, 2022 and April 20, 2023.

**Intervention:**

We designed a CDS questionnaire in the electronic medical record (EMR) to guide appropriate ordering of the GI panel. A “soft stop” reminder at the point of ordering prompted providers to confirm five appropriateness criteria: 1) documented diarrhea, 2) no recent receipt of laxatives, 3) *C. difficile* is not the leading suspected cause of diarrhea, 4) time period since a prior test is >14 days or prior positive test is >4 weeks and 5) duration of hospitalization <72 hours. The CDS was implemented in November 2022.

**Results:**

Compared to the pre-implementation period (*n* = 136), fewer tests were performed post-implementation (*n* = 92) with an IRR of 0.61 (*p* = 0.003). Inappropriate ordering based on laxative use or undocumented diarrhea decreased (IRR 0.37, *p* = 0.012 and IRR 0.25, *p* = 0.08, respectively). However, overall inappropriate ordering and outcome measures did not significantly differ before and after the intervention.

**Conclusions:**

Implementation of CDS in the EMR decreased testing and inappropriate ordering based on use of laxatives or undocumented diarrhea. However, inappropriate ordering of tests overall remained high post-intervention, signaling the need for continued diagnostic stewardship efforts.

## Introduction

Commercially available multiplex gastrointestinal polymerase chain reaction (PCR) testing panels (hereafter, “GI panels”) detect bacterial, viral, and parasitic pathogens.^1^ In validation studies, the sensitivity and specificity are high, >90% and >97%, respectively.^2,3^ Compared with standard stool cultures, testing with the GI panels has been associated with higher positive yield, reduced antibiotic use, decreased time to initiation of optimal antibiotic therapy, decreased length of hospital stay, and fewer diagnostic procedures.^4–7^

Despite these benefits, GI panels are subject to overuse. Pathogens of lower prevalence have been shown to have high false-positive rates.^8^ Additionally, there is low yield to GI panel testing after 72 hours of hospitalization^9^ because of low pre-test probability for infectious etiology of diarrhea in this setting.^10^ Further, there is low utility of repeat testing within 4 weeks of a positive test, as follow-up tests often redemonstrate the initial pathogen due to residual genetic material or colonization.^11,12^ To improve use of GI panels, ordering is commonly aimed at patients with documented diarrhea and without laxative use in the preceding 24–48 hours.^13,14^ Ensuring appropriate use of GI panels is important^15^ since inappropriate diagnosis and treatment is associated with risk for adverse drug events, development of antimicrobial resistance, and prolonged length of stay in hospitalized patients.^16^

The objective of this study was to assess the impact of a clinical decision support (CDS) intervention designed to facilitate appropriate use of a GI panel within a Veteran’s Affairs (VA) Healthcare System. The primary aim was to evaluate the frequency and appropriateness of GI panel ordering before and after implementation of the CDS. The secondary aim was to evaluate the impact of the CDS on select outcome quality measures.

## Methods

### Study population

The VA Maryland Healthcare System (VAMHCS) delivers healthcare services at 8 locations in 15 counties providing medical, surgical, rehabilitative, primary, mental health, and long-term care, in inpatient and outpatient settings. All GI panel tests completed in the VAMHCS between June 22, 2022 and April 20, 2023 were included.

### BioFire FilmArray GI Panel

The BioFire® FilmArray® GI panel (BioFire Diagnostics, Salt Lake City, UT), tests for the following pathogens; *Campylobacter* spp*. (C. jejuni, C. coli, C. upsalensis), C. difficile, Plesiomonas shigelloides, Salmonella, Vibrio* spp*. (V. parahaemolyticus, V. vulnificus, V. cholerae), Yersinia enterocolitica,* Enteroaggregative *E. coli* (EAEC), Enteropathogenic *E. coli* (EPEC), Enterotoxigenic *E. coli*, Shiga-like toxin-producing *E. coli*, *Shigella*/Enteroinvasive *E. coli*, *Cryptosporidium, Cyclospora cayetanensis,* Adenovirus, Astrovirus, Norovirus, Rotavirus, Sapovirus, *Entamoeba histolytica,* and *Giardia lamblia*. At the time of GI panel implementation, the facility suppressed five pathogens from reporting as treatment is typically not recommended for these pathogens of uncertain clinical significance. The suppressed results included EAEC and EPEC, which are likely to be GI colonizing flora; *Plesiomonas shigelloides,* which is more common in international travelers, and typically not treated unless severe^17^; and Astrovirus and Sapovirus, which are typical pathogens in children but not in the VA patient population. The suppressed pathogens may be released with diagnostic stewardship team oversight if requested by the ordering provider.

**Figure 1.**
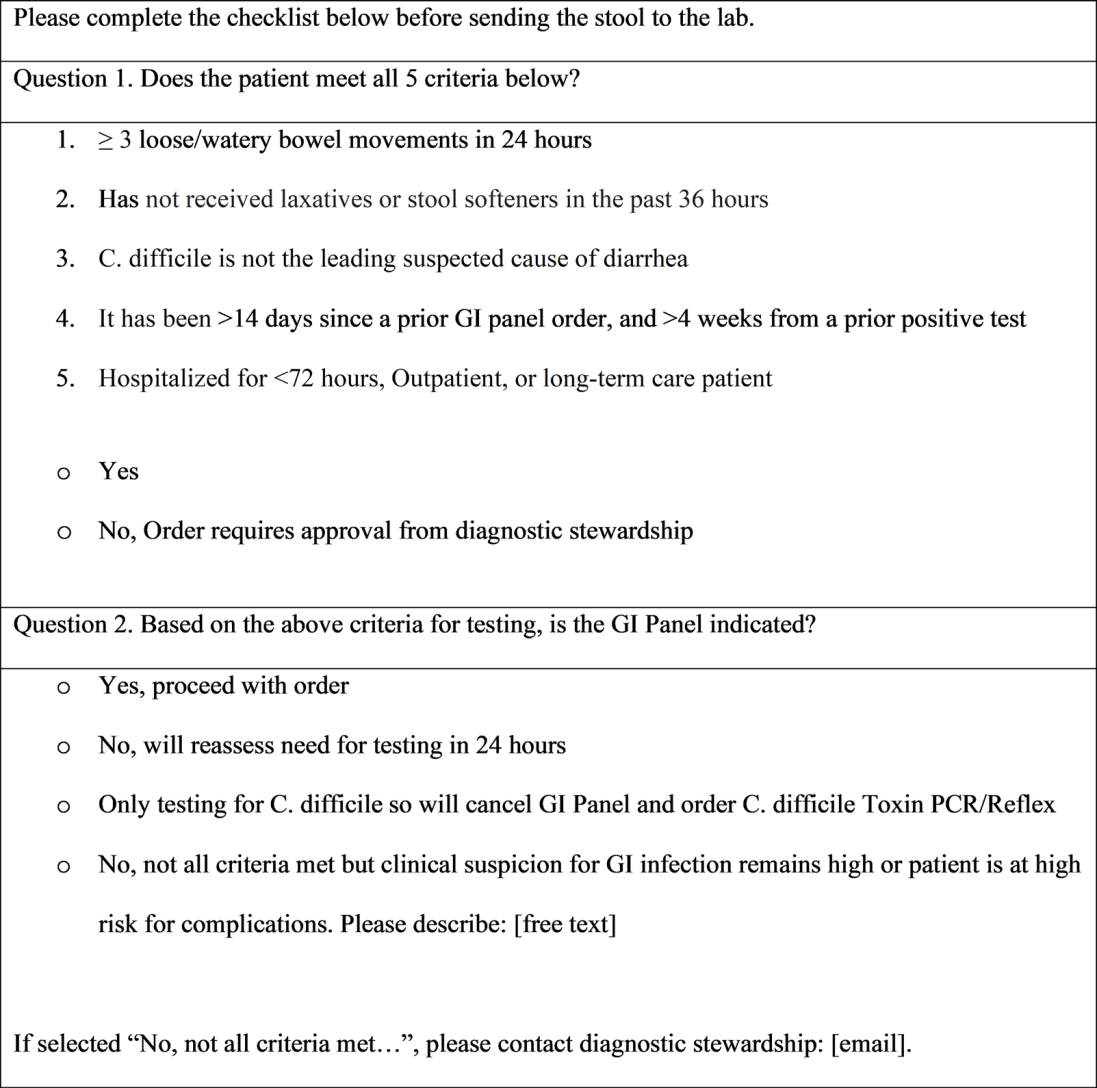
GI panel ordering questionnaire.

The GI panel was made available for ordering on June 22, 2022. A clinical decision support (CDS) questionnaire was developed in the VAMHCS EMR with input from Infectious Disease, Infection Control and Antimicrobial Stewardship, and launched November 16, 2022. Upon launch of the CDS, a facility-wide email instructed providers on use of the CDS and changes to the ordering process. The CDS was designed to guide appropriate ordering at the time of order placement by prompting providers to confirm that the patient met five criteria (Figure 1): 1) presence of diarrhea (≥3 loose or watery stools within 24 hours), 2) no receipt of laxatives or stool softeners within the antecedent 36 hours, 3) *C. difficile* is not the leading suspected cause of diarrhea, 4) time period since a prior test is >14 days or prior positive test is >4 weeks, and 5) duration of hospitalization is <72 hours. If all five criteria were not met, the provider was asked to either cancel the order or document a reason for proceeding and contact the diagnostic stewardship team. The CDS was designed as a “soft stop” at the point of provider orders, meaning that the questionnaire responses did not prevent ordering of the GI panel, but the questions must be answered to proceed to the lab order.

### Data collection and analysis

We selected diagnostic stewardship measures according to the literature described above based on appropriate use of the GI panel and selected established quality measures of appropriate antibiotic use (Table 2).^4,9,12,13,18,19^

With the support of laboratory informatics, Structured Query Language was used to query the Corporate Data Warehouse for patients on whom a GI panel was ordered. These patients were compared pre-intervention and post-intervention. Variables abstracted included patient demographics and primary diagnosis. Process measures included the number of inappropriate orders and inappropriateness according to the following criteria: placed after 72 hours of hospitalization, within 14 days of a prior test, within 4 weeks of a prior positive test, on stool softeners or laxatives within 36 hours prior to ordering, a concomitant order for standalone *C. difficile* PCR, or without documented diarrhea. Appropriateness indications were verified via chart review. Outcome measures included hospital length of stay (LOS) in days,^4^ mortality,^18^ and unnecessary antibiotic therapy for **≥**5 days without a confirmed bacterial infection.^19^

We compared the pre- and post-CDS groups using Chi square and one-way ANOVA tests. We then performed a bivariate binomial regression on process and outcome measures to calculate an incidence rate ratio (IRR) comparing pre- and post-intervention rates, using two-week periods as the unit of analysis. Data abstracted from the EMR was entered into a de-identified database and analyses were performed using SAS Studio (SAS Institute Inc., Cary, NC). Data were abstracted independently by two study team members and any discrepancies were reconciled.

This study was considered completed Quality Improvement work by the University of Maryland Baltimore Institutional Review Board.

## Results

**Figure 2.**
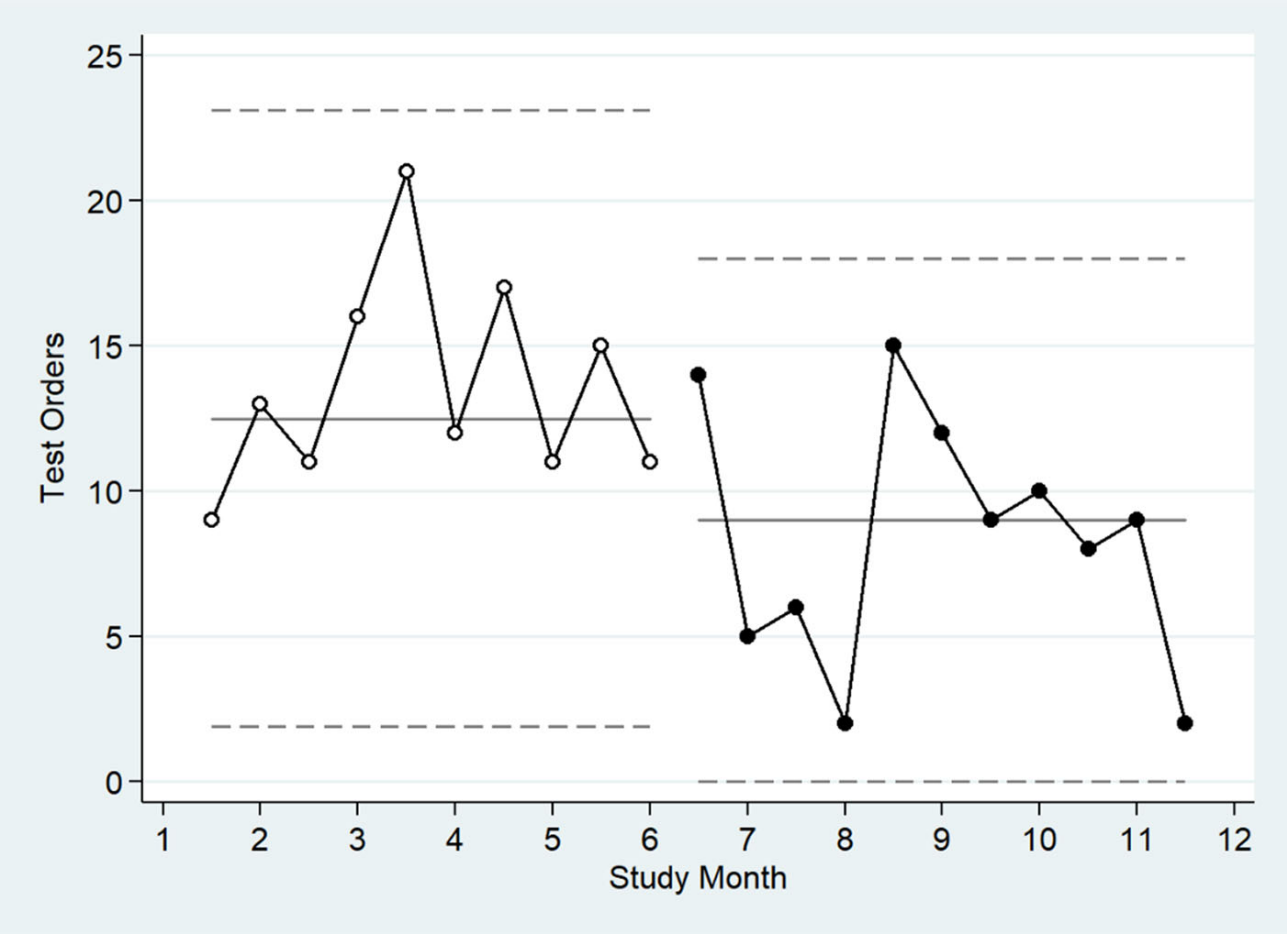
Chart of gastrointestinal polymerase chain reaction (PCR) panel ordering during study period, June 2022 to April 2023.

Patients on whom GI panel orders were completed during the study period were mostly male (80.2%) and mean age was 60.2 years (Table 1). Between June 22, 2022 and April 20, 2023, 228 GI panel orders were completed. During the pre-CDS period (June 22, 2022 to November 15, 2022), 136 orders were completed, and of these, 24 tests (17.7%) were positive for any pathogen. During the post-CDS period (November 16, 2022 to April 20, 2023), 92 orders were completed, with 23 (25.0%) returning positive for any pathogen, and four tests were positive for more than one pathogen (8.5%). A total of 9 distinct pathogens were identified, with *C. difficile* (*n* = 20) and Norovirus (*n* = 12) being the most frequent (Table 1). Orders were most frequently placed in outpatient settings (51.8%) followed by inpatient settings (30.3%). Frequency of GI panel orders placed after introduction of the order panel on June 22, 2022 peaked in August 2022, and rates decreased after introduction of the CDS in November 2022 (Figure 2). The mean LOS among hospitalized inpatients (*n* = 69) was 8.8 (SD 14.0) and did not differ between the pre- and post-CDS periods. Unnecessary antibiotic use and inpatient mortality were rare, limiting the ability to compare between pre- and post-CDS groups. Only one hospitalization, in the post-CDS group, ended in mortality. For two orders, antibiotics were continued for **≥**5 days despite a negative GI panel. Both of these orders were among patients in the pre-CDS period, and progress notes indicated continued suspicion of infectious diarrhea despite negative GI panel.

**Table 1.** Characteristics of patients with GI panel orders in study period^a^

Characteristic	Total *N* = 228	Pre-CDS *N* = 136	Post-CDS *N* = 92
**Gender, male,** ***n*** **(%)**	182 (80.2)	111 (82.2)	71 (77.2)
**Age, years**			
Mean (SD)	60.2 (15.1)	61.6 (14.8)	58.0 (15.4)
**Ordering site,** ***n*** **(%)**			
Outpatient	118 (51.8)	68 (50.0)	50 (54.4)
Inpatient	69 (30.3)	38 (27.9)	31 (33.7)
Emergency department	18 (7.9)	11 (8.1)	7 (7.6)
Residential^b^	23 (10.1)	19 (14.0)	4 (4.4)
**Pathogens identified,** ***n*** **(%)**	47 (20.6)	24 (17.7)	23 (25)
*Campylobacter (jejuni, coli, upsalensis)*	9 (17.3)	4 (16.7)	5 (17.9)
*C. difficile*	20 (38.5)	9 (37.5)	11 (39.3)
*Salmonella*	1 (1.9)	1 (4.2)	0
*Yersinia enterocolitica*	2 (3.8)	0	2 (7.1)
Shigella/Enteroinvasive *E. coli* (EIEC)	1 (1.9)	0	1 (3.6)
Enterotoxigenic *E. coli* (ETEC)	5 (9.6)	3 (12.5)	2 (7.1)
Norovirus	12 (23.1)	7 (29.2)	5 (17.9)
*Entamoeba histolytica*	1 (1.9)	0	1 (3.6)
*Giardia lamblia*	1 (1.9)	0	1 (3.6)
**Outcome measures,** ***n*** **(%)**			
Hospital LOS in days^c^, mean (SD)	8.8 (14.0)	8.2 (12.1)	9.5 (16.1)
Outcome of hospitalization – mortality^c^	1 (1.4)	0	1 (3.2)
Patients receiving unnecessary antibiotic treatment	2 (0.9)	2 (1.5)	0

a All *p*-values for comparison of Pre-CDS group and Post-CDS group characteristics were non-significant (*p* > .05), using Chi-Square for categorical characteristics and one-way ANOVA for age and hospital LOS.

b Includes patients in community living centers, residential treatment or recovery programs.

c Among inpatients only (excludes residential, outpatient, and emergency department); total *n* = 69, pre-CDS *n* = 38, post-CDS *n* = 31.

**Table 2.** Effect of Clinical Decision Support Tool (CDS) on GI panel ordering characteristics

Characteristic or Measure	Total	Pre-CDS	Post-CDS	IRR^a^ for CDS	95% CI	*p*-value*
**Total testing**	228	136	92	0.61	0.45, 0.85	**0.003**
**Test positivity,** ***n*** **(%)**	47 (20.6)	24 (17.7)	23 (25.0)	0.87	0.49, 1.54	0.637
**Process Measures,** ***n*** **(%)**						
Inappropriate testing	112 (49.1)	66 (48.5)	46 (50.0)	0.63	0.39, 1.02	0.059
After 72 hours hospitalization	12 (5.3)	7 (5.2)	5 (5.4)	0.65	0.21, 2.04	0.461
Within 14 days of a prior test	3 (1.3)	1 (0.7)	2 (2.2)	1.82	(0.16, 20)	0.625
Within 4 weeks of a prior positive test	4 (1.8)	1 (0.7)	3 (3.3)	2.72	0.28, 26.2	0.385
Patient received laxatives or stool softeners within prior 36 h	31 (13.6)	22 (16.2)	9 (9.8)	0.37	0.17, 0.81	**0.012**
Concomitant order for *C. diff* PCR test	76 (33.3)	42 (30.9)	34 (37.0)	0.74	0.45, 1.20	0.220
Without documented diarrhea	14 (6.2)	11 (8.2)	3 (3.3)	0.25	0.051, 1.20	0.084
Appropriate testing	116 (50.9)	70 (51.5)	46 (50.0)	0.61	0.40, 1.54	**0.012**

Note. *P*-values represent the probability that the observed incidence rate ratio estimated from negative binomial regression would have been observed by chance, with the null hypothesis being that IRR = 1. Though cumulative frequencies and proportions from the pre-intervention and post-intervention periods are reported in this table, negative binomial regression included data-points from 10 distinct pre-intervention two-week periods and 11 distinct post-intervention two-week periods. *Bold denotes statistical significance at *p* < 0.05 level.

a Incidence Rate Ratio, compared to the pre-CDS group.

Table 2 displays the comparison of GI panel orders placed during the pre-CDS period with the post-CDS period. About half of orders placed during the entire study period were inappropriate. The overall testing rate decreased throughout the study period (IRR 0.61, 95% CI 0.45, 0.85), with similar effects for both appropriate and inappropriate testing. The rate of test positivity did not significantly change throughout the study period (IRR 0.87, 95% CI 0.49, 1.54). The proportion of inappropriate orders differed by ordering site (*p* < .001 for Fisher’s exact test). The majority of orders placed in the Emergency Department (83.3%) or inpatient (65.2%) were inappropriate, compared to 38.1% and 30.4% for outpatient and residential, respectively (data not shown). The most common inappropriate ordering practice was concomitant *C. difficile* PCR ordering, 76/228 (33.3%). The next most common inappropriate ordering practice was on patients who had received laxatives or stool softeners within 36 hours prior to testing, 31/228 (13.6%) of all orders during the study period. Inappropriate testing of patients on laxatives decreased significantly in the post-CDS period compared to the pre-CDS period (IRR 0.37, *p* = 0.012). Similarly, inappropriate testing among patients without documented diarrhea decreased during the study period and trended toward statistical significance (IRR 0.25, *p* = 0.084). Orders were rarely placed on patients with a GI panel test within the last two weeks (1.3%) or prior positive test within the last four weeks (1.8%).

## Discussion

We analyzed 228 GI panel tests completed at a large VA healthcare system that included inpatient, outpatient, emergency department, and residential settings. The number of total GI panels ordered decreased from 136 before CDS implementation to 92 after implementation, consistent with a 39% reduction in ordering (IRR 0.61, *p* = 0.003). Ordering decreased for both inappropriate and appropriate tests so may reflect a general lack of awareness on how to order the test following the change in ordering practice. However, the inappropriate testing among patients on laxatives decreased significantly following introduction of the CDS (IRR 0.37, *p* = 0.012), and inappropriate testing for patients without documented diarrhea also trended toward significance (IRR 0.25, *p* = 0.084). This suggests that the “soft stop” advice provided via CDS positively impacted the decision to order a GI panel. A “hard stop” requiring the approval of a diagnostic stewardship consultant prior to release for orders that did not meet all five appropriateness criteria could further improve ordering practices and may be considered in subsequent CDS iterations.

Half of GI panel orders completed during the study period met at least one criterion for inappropriate ordering. While high, this is lower than the 61% prevalence of inappropriate ordering reported by O’Neal et al. in their assessment of GI panels ordered at a community teaching hospital.^13^ The primary reason for inappropriate ordering in our study was concomitant *C. difficile* PCR test (33.3%), similar to O’Neal et al. The CDS did not clarify that the GI panel included *C. difficile* testing, and additional provider education may decrease duplicate ordering going forward. Further, as our lab canceled concurrent *C. difficile* requests, duplicate orders did not result in duplicate tests.

During the study period overall, 20.6% of tests returned positive for any pathogen, which is lower than the 29%–35% reported by other studies.^5,6,13,20^ Additionally, the rate of co-detection of multiple pathogens in our sample was 8.5%, which is also lower than the 16%–28% reported by other studies.^4,21,22^ While not an a priori outcome of this study, it was noted that tests ordered after the introduction of the CDS had a higher positive yield (25.0%) than tests ordered pre-CDS (17.7%). Although the differences were not statistically significant, the trend suggests an improvement in pre-test probability that the patients on whom tests were ordered truly did have disease.

The strengths of this study include its focus on a VA healthcare system including GI panel orders placed across a range of ordering settings, which provides a representative snapshot of the appropriateness of GI panel ordering practices in acute care, long-term care, and ambulatory care. This population also tends to have more chronic comorbidities and socioeconomic barriers to care than non-veteran adults in the US,^23,24^ so this study lends insight into the impact of diagnostic stewardship measures in a unique population. Additionally, the age profile of this population (large proportion >65 years) likely reflects that of US adults who experience the highest burden of hospitalizations and deaths from acute gastroenteritis compared to younger adult populations with fewer comorbidities.

The study has limitations including relatively short study period (5 months pre-intervention and 5 months post-intervention) and small sample size. As this was a convenience sample, power calculations, and sample sizes were not pre-determined. Information about provider experience with the CDS was limited, such as how often they might have canceled the order or why clinicians chose to override the CDS. The current EMR system does not allow for interactive choice architecture which could have included population-specific risk factors and refined criteria based on clinical setting that could provide more patient-specific clinical decision support. Additionally, the retrospective study design and descriptive nature of the analyses prevent us from attributing process and outcome measures to the CDS.

In conclusion, additional diagnostic stewardship efforts including provider education and continued surveillance are needed. Given that over half of orders were placed in the outpatient setting, a focus on reducing inappropriate ordering among ambulatory providers could guide future interventions such as limiting the use of the GI panel as part of the diagnostic work-up for chronic diarrhea. We observed outpatients with chronic diarrhea of several years’ duration had GI panels ordered as part of the work-up to rule out infection, which is likely low yield.

In this retrospective analysis of GI panel orders completed at a large VA healthcare system, after implementation of a clinical decision support tool at the point of test ordering, fewer tests were ordered, and the positive yield of tests increased. Inappropriate ordering of the GI panel for patients on laxatives or stool softeners, or without documented diarrhea, decreased. These changes demonstrate the low-cost, high value of this simple intervention. However, the proportion of inappropriate tests ordered remained high, signaling the need for continued diagnostic stewardship to optimize the use of the GI panel.

## References

[1] Gastrointestinal (GI) pathogen panel | biofire diagnostics. https://www.biofiredx.com/products/the-filmarray-panels/filmarraygi/. Accessed October 29, 2022.

[2] Buss SN , Leber A , Chapin K , et al. Multicenter evaluation of the biofire filmarray gastrointestinal panel for etiologic diagnosis of infectious gastroenteritis. J Clin Microbiol 2015;53:915–925. doi: 10.1128/JCM.02674-14 25588652 PMC4390666

[3] Khare R , Espy MJ , Cebelinski E , et al. Comparative evaluation of two commercial multiplex panels for detection of gastrointestinal pathogens by use of clinical stool specimens. J Clin Microbiol 2014;52:3667–3673. doi: 10.1128/JCM.01637-14 25100818 PMC4187753

[4] Torres-Miranda D , Akselrod H , Karsner R , et al. Use of BioFire FilmArray gastrointestinal PCR panel associated with reductions in antibiotic use, time to optimal antibiotics, and length of stay. BMC Gastroenterol 2020;20:1–7. doi: 10.1186/s12876-020-01394-w PMC7392718 32727381

[5] Beal SG , Tremblay EE , Toffel S , Velez L , Rand KH. A gastrointestinal PCR panel improves clinical management and lowers health care costs. J Clin Microbiol 2018;56:1–9. doi: 10.1128/JCM.01457-17 PMC5744222 29093106

[6] Axelrad JE , Freedberg DE , Whittier S , Greendyke W , Lebwohl B , Green DA. Impact of gastrointestinal panel implementation on health care utilization and outcomes. J Clin Microbiol 2019;57:1–8. doi: 10.1128/JCM.01775-18 PMC6425162 30651393

[7] Baghdadi JD , Coffey KC , Leekha S , Johnson JK , Diekema DJ , Morgan DJ. Diagnostic Stewardship for Comprehensive Gastrointestinal Pathogen Panel Tests. Curr Infect Dis Rep 2020;22:11908. doi: 10.1007/s11908-020-00725-y

[8] Hitchcock MM , Hogan CA , Budvytiene I , Banaei N. Reproducibility of positive results for rare pathogens on the FilmArray GI Panel. Diagn Microbiol Infect Dis 2019;95:10–14. doi: 10.1016/j.diagmicrobio.2019.03.013 31029490 PMC7286436

[9] Hitchcock MM , Gomez CA , Banaei N. Low yield of FilmArray GI panel in hospitalized patients with diarrhea: An opportunity for diagnostic stewardship intervention. J Clin Microbiol 2018;56:1–7. doi: 10.1128/JCM.01558-17 PMC5824048 29237784

[10] Tirlapur N , Puthucheary ZA , Cooper JA , et al. Diarrhoea in the critically ill is common, associated with poor outcome, and rarely due to Clostridium difficile. Sci Rep 2016;6:1–9. doi: 10.1038/srep24691 27094447 PMC4837391

[11] Scallan E , Griffin PM , Angulo FJ , Tauxe R V. , Hoekstra RM. Foodborne illness acquired in the United States—unspecified agents. Emerg Infect Dis 2011;17:16. doi: 10.3201/EID1701.P21101 21192849 PMC3204615

[12] Park S , Hitchcock MM , Gomez CA , Banaei N. Is follow-up testing with the filmarray gastrointestinal multiplex PCR panel necessary? J Clin Microbiol 2017;55:1154–1161. doi: 10.1128/JCM.02354-16 28122874 PMC5377843

[13] O’Neal M , Murray H , Dash S , Al-Hasan MN , Justo JA , Boostraver PB. Evaluating appropriateness and diagnostic stewardship opportunities of multiplex polymerase chain reaction gastrointestinal testing within a hospital system. Ther Adv Vaccines 2020;7:1–10. doi: 10.1177/2049936120959561 PMC7513010 33014363

[14] Howard-Anderson JR , Sexton ME , Robichaux C , et al. The impact of an electronic medical record nudge on reducing testing for hospital-onset Clostridioides difficile infection. Infect Control Hosp Epidemiol 2020;41:411–417. doi: 10.1017/ICE.2020.12 32036798 PMC7909614

[15] Shane AL , Mody RK , Crump JA , et al. 2017 Infectious diseases society of America clinical practice guidelines for the diagnosis and management of infectious diarrhea. Clin Infect Dis An Off Publ Infect Dis Soc Am 2017;65:e45. doi: 10.1093/CID/CIX669 PMC5850553 29053792

[16] 2019 Antibiotic Resistance Threats Report | CDC. https://www.cdc.gov/drugresistance/biggest-threats.html#cdiff. Accessed October 30, 2022.

[17] Michael Janda J , Abbott SL , McIver CJ. Plesiomonas shigelloides revisited. Clin Microbiol Rev 2016;29:349–374. doi: 10.1128/CMR.00103-15 26960939 PMC4786884

[18] Morris AM. Antimicrobial stewardship programs: appropriate measures and metrics to study their impact. Curr Treat Options Infect Dis 2014;6:101–112. doi: 10.1007/s40506-014-0015-3 25999798 PMC4431704

[19] Arcenillas P , Xercavins M , March P , et al. Assessment of quality indicators for appropriate antibiotic use. Antimicrob Agents Chemother 2018;62:1–9. 10.1128/AAC.00875-18 PMC6256780 30249698

[20] Cybulski RJ , Bateman AC , Bourassa L , et al. Clinical impact of a multiplex gastrointestinal polymerase chain reaction panel in patients with acute gastroenteritis. Clin Infect Dis 2018;67:1697–1704. doi: 10.1093/CID/CIY357 29697761

[21] Spina A , Kerr KG , Cormican M , et al. Spectrum of enteropathogens detected by the FilmArray GI Panel in a multicentre study of community-acquired gastroenteritis. Clin Microbiol Infect 2015:719–728. doi: 10.1016/j.cmi.2015.04.007 25908431

[22] Piralla A , Lunghi G , Ardissino G , et al. FilmArray^TM^ GI panel performance for the diagnosis of acute gastroenteritis or hemorragic diarrhea. BMC Microbiol 2017;17:1–10. doi: 10.1186/s12866-017-1018-2 28494766 PMC5427568

[23] Trivedi AN , Grebla RC , Wright SM , Washington DL. Despite improved quality of care in the Veterans Affairs health system, racial disparity persists for important clinical outcomes. Health Aff (Millwood) 2011;30:707–715. doi: 10.1377/HLTHAFF.2011.0074 21471492

[24] Jha AK. Learning from the past to improve VA health care. JAMA 2016;315:560–561. doi: 10.1001/JAMA.2016.0243 26864409

